# Powerful *p*-value combination methods to detect incomplete association

**DOI:** 10.1038/s41598-021-86465-y

**Published:** 2021-03-26

**Authors:** Sora Yoon, Bukyung Baik, Taesung Park, Dougu Nam

**Affiliations:** 1 Department of Biological Sciences, Ulsan National Institute of Science and Technology, Ulsan, 44919 Republic of Korea; 2 Department of Statistics, Seoul National University, Seoul, 08826 Republic of Korea; 3 Interdisciplinary Program in Bioinformatics, Seoul National University, Seoul, 08826 Republic of Korea; 4 Department of Mathematical Sciences, Ulsan National Institute of Science and Technology, Ulsan, 44919 Republic of Korea

**Keywords:** Computational biology and bioinformatics, Mathematics and computing

## Abstract

Meta-analyses increase statistical power by combining statistics from multiple studies. Meta-analysis methods have mostly been evaluated under the condition that all the data in each study have an association with the given phenotype. However, specific experimental conditions in each study or genetic heterogeneity can result in “unassociated statistics” that are derived from the null distribution. Here, we show that power of conventional meta-analysis methods rapidly decreases as an increasing number of unassociated statistics are included, whereas the classical Fisher’s method and its weighted variant (wFisher) exhibit relatively high power that is robust to addition of unassociated statistics. We also propose another robust method based on joint distribution of ordered *p-*values (ordmeta). Simulation analyses for *t*-test, RNA-seq, and microarray data demonstrated that wFisher and ordmeta, when only a small number of studies have an association, outperformed existing meta-analysis methods. We performed meta-analyses of nine microarray datasets (prostate cancer) and four association summary datasets (body mass index), where our methods exhibited high biological relevance and were able to detect genes that the-state-of-the-art methods missed. The metapro R package that implements the proposed methods is available from both CRAN and GitHub (http://github.com/unistbig/metapro).

## Introduction

Meta-analysis is used to aggregate summary statistics (effect sizes, standard errors, or *p-*values) obtained from different studies that share the same null hypothesis^1–3^. Meta-analysis usually increases statistical power because it combines the signals of moderate significance in each study, while false positives are kept controlled. Thus, meta-analysis has been widely applied to medical and genetic research, such as identifying differentially expressed genes in transcriptome analysis, finding causal variants in genome-wide association studies (GWAS), and assessing prognostic features for clinical trials^4–6^. Although meta-analysis methods that combine *p*-values or *Z*-scores have widely been used before, they were not able to address the effect size and heterogeneity in each study. For instance, even a small effect size in a dataset could lead to a significant *p*-value if a large sample size was used. Therefore, current meta-analysis makes use of methods that combine effect sizes in most cases. However, methods that combine *p-*values are still being used in genetic or systems biology study when analyzing low-frequency or rare variants when effect sizes are not available, or when results of different types of experiments need to be combined^2,7,8^.

Many *p*-value or *Z*-score combining methods including Fisher’s method take the null hypothesis that the true effect in each of the combined datasets is zero^2^. This suggests a high sensitivity of the methods even when only a subgroup of the combined datasets have a nonzero effect size. We call this condition as *incomplete association*. We paid attention to this null hypothesis and compared the performance of various *p*-value, *Z*-score, effect size-based meta-analysis methods. We found that the classical Fisher’s method outperformed other methods in detecting such incomplete association. Such an advantage of the *p*-value combination method shows a great potential to discover novel markers or differentially expressed genes that the commonly used methods miss.

Here, we propose two *p*-value combination methods that are able to detect incomplete associations better than existing methods. To the authors’ knowledge, methods that tackle the incomplete association that is detectable in neither individual study nor conventional meta-analysis have rarely been investigated. We call a meta-analysis method is *robust* if it is able to detect the incomplete association even when a majority of the datasets combined have zero effects. Hereafter, *associated p-value* (or *p1*-value) denotes the *p-*value obtained from the experiment with a positive (or negative) effect, and *unassociated p-value* (or *p0*-value), the uniformly distributed under the null hypothesis.

We propose a generalized Fisher’s method weighted by sample-sizes (denoted as wFisher). wFisher holds not only the high robustness of the original Fisher’s method, but also the high power of weighted methods. In addition, we propose another robust method that uses the minimum of marginal *p-*values in joint distribution of ordered *p-*values (denoted as ordmeta). The proposed methods use only *p*-values and their directionalities but outperformed the state-of-the-art meta-analysis methods in detecting incomplete associations for simulated *t*-tests, and simulated RNA-seq and microarray data. Further, our methods were applied to meta-analysis of prostate cancer microarray data and body mass index (BMI) association data, where they exhibited high biological relevance and were able to detect many genes missed by other meta-analysis methods.

## Methods

### *p*-value or *Z*-score combination methods

More than a dozen methods to combine *p-*values have been developed thus far. The first one developed by Fisher used $\chi^{2}$-distribution^9^ (denoted as Fisher’s method). Lancaster generalized Fisher’s method by assigning different weights to degrees of freedom (DFs) of each study^10^ (denoted as Lancaster’s method). Stouffer further developed the method by using inverse normal distribution (denoted as *Z-*method)^11^, and its generalized version that weighted each experiment by sample sizes was also developed^12^ (denoted as weighted *Z-*method). The weighted *Z-*method exhibited increased power compared to other *p*-value or *Z*-score combining methods^13^. A similar high power was observed for Lancaster’s method when each experiment was weighted by sample sizes^14^. Because of this advantage, weighted methods have been strongly preferred despite the arguments against using weights in meta-analysis^15^. The high power of weighted methods has been demonstrated with each individual experiment involving identically distributed effect sizes. However, this condition is often not met in real situations because of the heterogeneity of the populations under consideration and the environmental and study-specific factors that lower the quality of data. For example, 25% of tagSNPs identified in European GWAS showed significantly different effect sizes in non-European ancestry population^16^. We found that the power of the weighted *Z*- and Lancaster’s methods rapidly decreases as an increasing number of unassociated *p*-values are combined, whereas the Fisher’s method holds a relatively high power. In particular, we found that the size of DFs assigned to individual experiments have a critical effect on the robustness of methods.

Let $p_{1} , \ldots , p_{n}$ be *p-*values obtained from *n* independent experiments where the model to generate *p*-values are specified correctly. Each *p-*value is assumed to have uniform distribution on the unit interval [0, 1]. Many existing methods to combine *p*-values hypothesize that none of the given *p*-values has an association. Thus, the hypotheses of interest are given as follows:

- H_0_: none of the given *p*-values $p_{1} , \; \ldots , \;p_{n}$ are associated with the phenotype.
- H_1_: one or more *p*-values are associated with the phenotype.

However, most comparative studies for meta-analysis methods have been conducted using simulated data where all the statistics (or *p*-values) have an association to test the statistical power.

The Fisher’s method has been the most commonly used to combine *p-*values^9^. The following test statistic *T* has $\chi^{2}$-distribution with DF of $2n$.

$$T = - 2\mathop \sum \limits_{i = 1}^{n} \log (p_{i} )\sim \chi^{2} (2n)$$

Then, the combined *p-*value is calculated as the right-tail probability ${\text{P}}_{{\chi^{2} (2n)}} (T > t)$, where *t* is the observed *T* value. One of the main limitations of Fisher’s method is that it assigns only the equal weight for each experiment. Lancaster’s method generalized Fisher’s method by assigning different weights using the additivity of $\chi^{2}$-distribution, and a special case with the weights of sample sizes was tested^10,14^. Let $s_{1} ,\; \ldots ,\; s_{n}$ be sample sizes for each dataset, and $S = \sum\nolimits_{j = 1}^{n} {s_{j} }$ be their summation. In Lancaster’s method, each $p_{i}$ is transformed to the $p_{i}$th quantile of chi-square distribution with $s_{i}$ DFs, $F_{{\chi^{2} (s_{i} )}}^{ - 1} (p_{i} )$, then $L = \sum\nolimits_{i = 1}^{n} {F_{{\chi^{2} (s_{i} )}}^{ - 1} (p_{i} ) \sim \chi^{2} (S)}$. By assigning different weights to each *p*-value, Lancaster’s method exhibited an increased power compared to Fisher’s method. However, it uses much larger total DFs than those of Fisher’s method. We demonstrate in “Results” that these large DFs in Lancaster’s method cause a drastic power decrease when only a small number of datasets have an association.

### Weighted Fisher’s method (wFisher)

We propose to use a gamma distribution to assign non-integer weights to each *p*-value that are proportional to sample sizes, while the total weight is kept as small as that of Fisher’s method (2*n*). Whereas the chi-square distribution uses only the integer DFs, its natural generalization (gamma distribution) accepts non-integer DFs. Our method, dubbed *wFisher*, exhibited both the high power of Lancaster’s method and the high robustness of Fisher’s method. Recall that $\chi^{2}$-distribution with ${\text{DF}} = 2n$ is a special case of gamma distribution $\Gamma (k,\;\theta )$ with shape parameter $k = n$ and scale parameter $\theta = 2$. Gamma distribution provides a great flexibility to assign non-integer weights. Let $F_{{X;k_{i} ,2}} (x)$ be gamma distribution function with $k = k_{i} = n \times s_{i} /S,$ $i = 1,\;2, \ldots ,n$, and $\theta = 2$. Note that $\sum\nolimits_{i = 1}^{n} {k_{i} = n}$. In the *Z*-score method, the individual *p*-value $p_{i}$ is transformed to a standard normal variable $\Phi^{ - 1} (p_{i} )$, and their sum, with or without weights, is used for the test statistic. Similarly, we transform each *p*-value to a gamma variable with designated (weighted) DFs. Then, the sum of those gamma variables, which also has a gamma distribution, becomes our test statistic. One difference is that the weights are reflected in DFs in our method, whereas the weights in the *Z*-score method are applied to the transformed normal variables.

The *p*-value, $p_{i}$ in each study is transformed to a gamma variable $X_{i} = F_{{X;k_{i} ,2}}^{ - 1} (p_{i} )$ with the parameters $k = k_{i}$ and $\theta = 2$. Then, their summation $X^{\prime} = X_{1} + X_{2} + \cdots + X_{n}$ (our test statistic) also follows a gamma distribution with $k = n$ and $\theta = 2$. Thus, for an observed summation $x^{\prime} = x_{1} + x_{2} \cdots + x_{n}$, the combined *p-*value (left-tail) is evaluated as follows:

$${\text{wFisher}}\;p {\text{ - value}} = P_{\Gamma (n,2)} (X^{\prime} < x^{\prime}) = P_{{\chi^{2} (2n)}} (X^{\prime} < x^{\prime}).$$

### A method based on joint distribution of order statistic (ordmeta)

Ordmeta is a novel *p-*value combination method using the joint distribution of ordered *p-*values. Let $p_{(r)}$ be the *r*th smallest among *n* independent *p-*values. These values follow joint distribution of ordered uniform random variables with beta marginal distribution as follows^17^:

$$p_{(r)} \sim Beta\;\left( {r, \;n - r + 1} \right)$$

**Figure 1:**
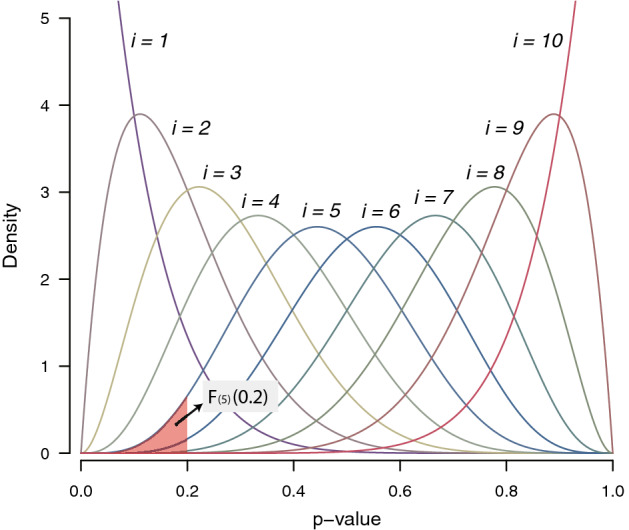
Beta marginal density functions for joint distribution of order statistic. For 10 input *p-*values obtained from independent experiments, the marginal density functions for *i*th *p-*value are shown. The shaded area represents 5th marginal *p-*value when 5th input *p-*value equals to 0.2.

Let $f_{(r)} (x)$ be the density function of $p_{\left( i \right)}$,

$$f_{(r)} (x) = \frac{1}{B(r, \;n - r + 1)}x^{r - 1} (1 - x)^{n - r}$$

where $B$ is beta function and $F_{(r)} (x)$ be its cumulative distribution function. Then, the *p-*value for each marginal distribution becomes $F_{(r)} (p_{(r)} )$ (Fig. 1). Song and Tseng^18^ used this marginal distribution of an order statistic to detect differentially expressed genes in meta-analyses of microarray data (denoted as *r*th ordered *P*-value (roP)). roP method used the marginal distribution of a fixed order (*r*) and the same distribution is applied to all genes. However, each study has a different group of differentially expressed genes, which means the optimal orders are different between genes. The main problem of roP is that the optimal order for each gene is not known. As will be seen in our simulation tests, the performance of roP largely varies depending on the selected value *r*. To address this problem, we propose a novel method, ordmeta that selects the smallest marginal *p*-value and evaluates its own *p*-value using joint distribution of order statistic. This approach adaptively selects the optimal order for each gene without any prior knowledge and evaluates the corresponding *p*-value.

Let $X$ be the minimum of the *n* marginal *p*-values.

$$X = \mathop {\min }\limits_{1 \le r \le n} F_{(r)} (p_{(r)} ).$$

The probability ${\text{P}}(X > \alpha )$ can be explicitly calculated using joint distribution of order statistic as follows:

$$\begin{aligned} {\text{P}}(X > \alpha ) & = {\text{P}}\left( {F_{(1)} (p_{(1)} ) > \alpha , \ldots ,F_{(n)} (p_{(n)} ) > \alpha } \right) \\ & = {\text{P}}\left( {p_{(1)} > F_{(1)}^{ - 1} (\alpha ), \ldots , p_{(n)} > F_{(n)}^{ - 1} (\alpha )} \right) \\ & = n! \mathop \smallint \limits_{{F_{\left( n \right)}^{ - 1} \left( \alpha \right)}}^{1} \ldots \mathop \smallint \limits_{{F_{\left( 2 \right)}^{ - 1} \left( \alpha \right)}}^{{t_{3} }} \mathop \smallint \limits_{{F_{\left( 1 \right)}^{ - 1} \left( \alpha \right)}}^{{t_{2} }} 1 dt_{1} \ldots dt_{n} . \\ \end{aligned}$$

Thus, the combined *p-*value is given as

$${\text{Ordmeta}}\;p{\text{ - value}} = {\text{P}}(X \le \alpha ) = 1 - {\text{P}}(X > \alpha ).$$

Ordmeta calculates *p-*value for the minimum marginal *p-*value. In other words, it assesses the positions of each marginal statistic $p_{(i)}$ to select the optimal one and assess its significance using joint distribution of order statistic. Thereby, the ordered *p*-values larger than the optimal $p_{(i)}$ have no effect on the final *p*-value, which confers the robustness. This optimal value also suggests that the *p*-values smaller than the optimal one are *p1*-values.

### Integrating two-tailed *p*-values

When individual *p*-values are obtained from two-tailed tests and effect directions are also given, those *p*-values are halved and synchronized according to the effect direction. In other words, each $p_{i}$ is converted to $p_{i} /2$ if the directions of true effects and integration coincide or to $1 - p_{i} /2$ otherwise, and integrate the resulting *p*-values. The integration is done once more for the complimentary *p*-values. The smaller of the two combined *p-*values is selected and multiplied by two for the two-tailed meta-analysis *p-*value.

### Simulation based on *t*-tests

**Figure 2:**
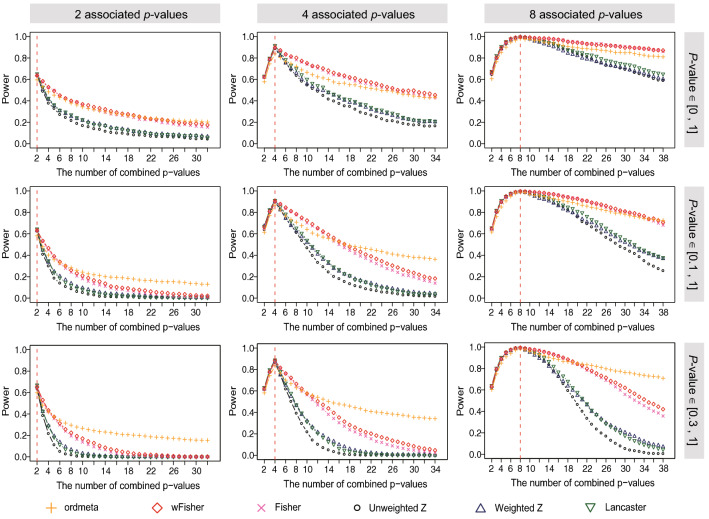
*t*-test simulation result. Statistical power of six *p-*value combining methods was tested for different numbers of associated *p*-values, unassociated *p-*values, and three different ranges of unassociated *p-*values. The vertical dashed line represents the boundary between associated and unassociated *p-*values. Up to the dashed line, only associated *p*-values were combined. To the right of dashed line, only unassociated *p-*values were added. In the first, second, and third rows of figures, unassociated *p-*values were sampled from intervals [0, 1], [0.1, 1], and [0.3, 1], respectively.

We simulated *t*-tests similar to those of previous works^13,14^ to compare power and false positive control for six *p-*value combining methods: wFisher, ordmeta, Fisher’s method, *Z-*method, weighted *Z-*method, and a Lancaster’s method with $DF_{i} = s_{i}$. The power was compared by including *p0-*values to a given set of *p1*-values. First, 2, 4, or 8 *p1*-values were combined and the corresponding powers were compared. Then, *p0-*values were also combined with the *p1*-values. A maximum of 30 *p0-*values were included. Standard normal values were sampled with sample sizes of 30–2000, and one sample *t*-test was performed for mean values of 0.03–0.1 to generate *p1*-values. *P0-*values were sampled from uniform distribution for one of the three ranges: [0.0, 1.0], [0.1, 1.0], and [0.3, 1.0]. Each scenario was repeated 1000 times (Fig. 2). To test false positive control, 2, 10, 30, and 100 *p-*values (sampled from [0, 1]) were generated and combined 100,000 times, and the proportion of results with combined *p-*values less than 0.05 was measured (Table 1).

**Table 1:** False positive control results for the significance cutoff 0.05.

Number of *p-*values	wFisher	ordmeta	*Z-*method	Weighted Z	Lancaster	Fisher
2	0.0492	0.0490	0.0492	0.0484	0.0486	0.0487
10	0.0499	0.0490	0.0506	0.0505	0.0512	0.0500
30	0.0497	0.0501	0.0496	0.0497	0.0491	0.0496
100	0.0504	0.0503	0.0498	0.0489	0.0493	0.0504

### Simulation of RNA-seq meta-analysis

**Figure 3:**
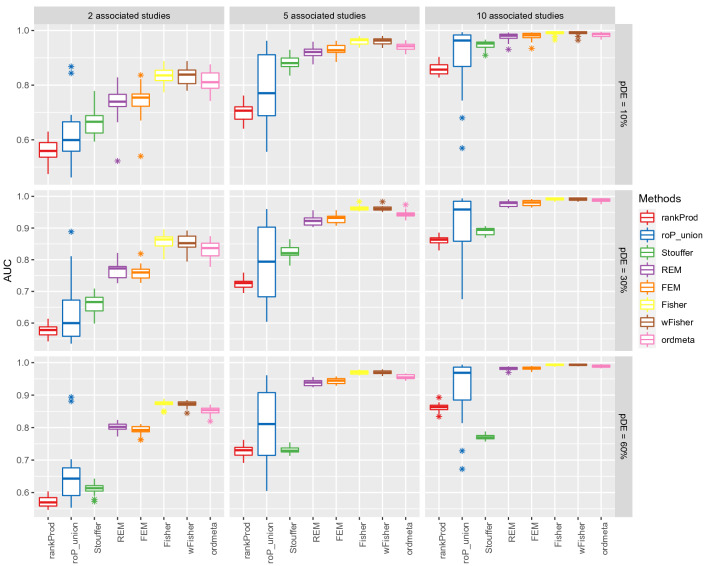
RNA-seq simulation results. Area under the ROC curves (AUCs) of eight meta-analysis methods were compared for different numbers of associated studies and significant genes. Among 20 studies used for the meta-analysis, 2, 5 and 10 studies were introduced as associated studies. The results with 10, 30 and 60% of significant genes are shown in the first, second, and third rows, respectively.

The RNA-seq data for TCGA Kidney Renal Clear Cell Carcinoma/normal dataset were downloaded from GDAC (http://gdac.broadinstitute.org). The mean and dispersion parameters of this dataset were estimated using edgeR package^19^. Using these parameter values, multiple RNA-seq datasets for 1000 genes were simulated using negative binomial distribution. 10%, 30%, or 60% genes were made differentially expressed (DE) with 1.3 or larger fold changes. See our paper^20^ for more detailed method to simulate read count data. The count data were then voom-transformed for moderated *t*-test (DE analysis)^21^. We simulated twenty RNA-seq datasets, where only two, five, or ten datasets included DE genes (incomplete association) and three different proportions of DE genes (10%, 30%, or 60%) were included in those datasets. Each simulation was repeated ten times and the results were represented as boxplots (Fig. 3). wFisher and ordmeta were implemented using our metapro package and six existing meta-analysis methods for gene expression data [rankProd^22^, roP^18^, Stouffer, Fisher, random effects model (REM)^23^ and fixed effects model (FEM)] were implemented using the metaDE R package^24^. Because the optimal order *r* of roP is not known in advance, it was implemented for all the even orders from two to 20 and all those results were combined to compare with other methods. The performance of meta-analysis methods was compared by their area under the receiver operating curve (AUC), true positive rate (TPR), and true false discovery rate (true FDR). The true FDR is the proportion of non-DE genes among the significant genes (*q*-value < 0.05) and indicates the extent of reliability of the predicted DE genes. We calculated true FDR only when five or more significant genes were detected in each method.

### Meta-analysis of microarray gene expression data

We analyzed the nine microarray datasets provided within the metaDE package, each containing both prostate cancer and normal samples. Because these datasets included three datasets with a low quality (“bad” datasets) as assessed by MetaQC package^25^, these data serve as a good example to test incomplete association.

The six datasets with a good quality (“good” datasets) were used to select true and false DE genes. After preprocessing the datasets using MetaDE package, 4436 genes commonly found in all the six datasets were used for meta-analysis. Among them, upregulated genes whose meta-analysis *q*-value is larger than 0.05 (less than 0.05) for at most one meta-analysis method were regarded as true upregulated DE genes (false upregulated DE genes). True and false downregulated genes were obtained similarly. These true and false DE genes served as gold standards to compare the performance of meta-analysis methods.

**Figure 4:**
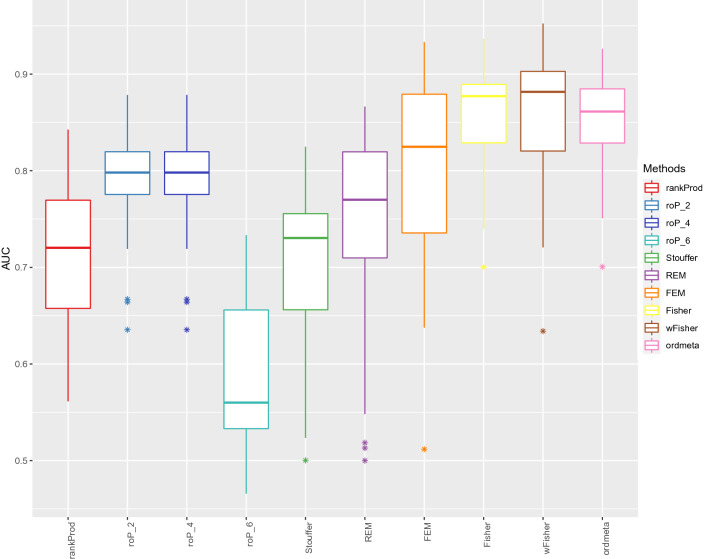
Microarray simulation results. Area under the ROC curves (AUCs) of ten meta-analysis methods were compared. The sample labels of seven out of the nine studies were permuted to make unassociated studies. roP was tested for three *r* parameters, 2, 4, and 6 denoted as (roP_2, roP_4, and roP_6, respectively).

Then, we analyzed all the nine datasets. After preprocessing the data, only 676 out of the 4436 genes remained. These genes included 161 and 234 true and false upregulated genes and 195 and 206 true and false downregulated genes, respectively. We selected two out of the six “good” datasets (15 cases in total). For the remaining four “good” datasets and the three “bad” datasets, we permuted the sample labels for each dataset, so that we expect no DE genes in these seven datasets. In other words, our test data represent an incomplete association where only two out of nine datasets include DE genes. We repeated the test for the 15 possible data combinations for both up and downregulation cases comprising 30 instances in total. The performance of the meta-analysis methods was analyzed by comparing the 30 AUCs, TPRs, and true FDRs between methods (Fig. 4).

We also analyzed the nine original datasets and compared the biological relevance of the meta-analysis results. Because all the REM DE genes were included in the FEM DE genes and Fisher and wFisher DE genes were similar to each other, we compared the results of only three meta-analysis methods (ordmeta, wFisher, and FEM) for simplicity. We downloaded the genes that belonged to the “Malignant neoplasm of prostate geneset” (C0376358) from DisGeNET database^26^, and used them as gold standard genes. The biological relevance of DE genes (FDR *q*-value < 0.01) was assessed by the enrichment (hypergeometric distribution) *p*-value and corresponding odds ratio (OR) of gold standard genes within DE genes as follows:

$${\text{Enrichment}}\;p{\text{ - value}} = 1 - \mathop \sum \limits_{i = 0}^{O - 1} \frac{{\left( {\begin{array}{*{20}c} G \\ i \\ \end{array} } \right)\left( {\begin{array}{*{20}c} {N - G} \\ {D - i} \\ \end{array} } \right)}}{{\left( {\begin{array}{*{20}c} N \\ D \\ \end{array} } \right)}}$$

$${\text{OR}} = \frac{{\left( {G \cap D} \right)}}{D}/\frac{G}{N}$$

where N is the total number of genes, D is the number of DE genes, and G is the number of gold standard genes. We used 635 genes (out of 676) that belonged to HUGO Gene Nomenclature Committee genes used in DisGeNET database to assess the biological relevance (Table 2).

**Table 2:** Comparison of biological relevance of significant genes. Nine prostate cancer microarray datasets were analyzed. Enrichment *p*-values and odds ratios of gold standard genes (C0376358) in differentially expressed genes for three meta-analysis methods are shown.

	*p*-value	Odds ratio	Gold standard genes	Significant genes
ALL	1	1	67	635
FEM	0.03946	1.55	57	467
wFisher	**0.00476**	**2.22**	56	442
ordmeta	0.00762	2.03	54	426
Union of three	0.01394	2.05	56	442

### Metapro R package and other meta-analysis tools

We developed an R package ‘metapro’ that implements wFisher and ordmeta methods as well as other *p-*value combining methods tested in this study. The package automatically imports ‘rSymPy’ package for the symbolic calculation of multiple integrals in ordmeta function. Metapro is available from both CRAN and GitHub (http://github.com/unistbig/metapro). For meta-analysis of RNA-seq and microarray data, we used the R packages metaDE^24^ and metaQC^25^. Individual expression dataset was analyzed using moderated *t*-test followed by meta-analysis pipelines provided by these R packages. MetaDE provides six meta-analysis methods, rankProduct, roP**,** Stouffer, Fisher, FEM, and REM for analyzing RNA-seq and microarray data. For the meta-analysis of BMI association data, weighted *Z-*method was implemented using METAL^27^ (https://genome.sph.umich.edu/wiki/METAL). Fixed and random effects models were implemented using GWAMA^28^ (https://www.geenivaramu.ee/en/tools/gwama). The random effects model developed by Han and Eskin^29^ (denoted as RE-HE) was implemented using METASOFT (http://genetics.cs.ucla.edu/meta_jemdoc/). MR-MEGA was implemented using MR-MEGA^30^ (https://www.geenivaramu.ee/en/tools/mr-mega).

## Results

### Comparison of *p-*value combining methods for simulated *t*-tests

We compared the performance of wFisher and ordmeta with that of existing *p-*value or *Z*-score combining methods (Fisher, *Z-*method, weighted *Z-*method, and Lancaster). To test the false positive control, 2, 10, 30, and 100 *p0-*values that were generated from one-tailed *t*-test with effect size zero and were combined using each method (Table 1). All the tested methods controlled the false positives well regardless of the number of combined *p-*values.

Next, the statistical power of the six methods was compared by combining 2, 4, or 8 *p1*-values, and was also compared by including *p0-*values (Fig. 2). The statistical power was measured by counting the instances with the combined *p-*value < 0.01 out of 1000 trials. When only *p1*-values were combined, all the six methods showed similar powers overall; the two weighted methods, Lancaster and wFisher exhibited slightly higher power as compared to unweighted methods, and ordmeta showed the lowest power. As *p0-*values were included, the power of Lancaster, *Z*-method, and weighted *Z-*method rapidly decreased, while Fisher, wFisher, and ordmeta exhibited a relatively slow decline. Notably, these results demonstrate (1) Fisher and *Z-*method are quite different methods, although the latter simply uses *Z*-scores transformed from *p*-values and (2) wFisher is superior to the original Fisher’s method, both with or without *p0-*values. Interestingly, another weighted Fisher’s method, Lancaster showed a rapid decline as opposed to Fisher or wFisher. Lancaster uses very large DFs (sample sizes), which make the individual chi-square distributions close to a normal distribution, while Fisher or wFisher use much smaller DFs.

In particular, ordmeta exhibited the best power when only a small number of associated *p-*values were included or some large *p0-*values (e.g., [0.1, 1] or [0.3, 1]) were combined. We also tested if ordmeta correctly predicts *p1*-values using the optimal *p-*value. For *t*-tests where half of the *p-*values were associated, the predicted *p1*-values exhibited high specificity with median 0.98–1.00 and decent sensitivity with median 0.5–0.6 for 10, 30, and 100 input *p-*values.

### Comparison of meta-analysis methods for simulated RNA-seq data

We compared the performance of two proposed methods (wFisher and ordmeta) and six existing meta-analysis methods for gene expression data by simulating RNA-seq data for the nine different scenarios of incomplete association: three different proportions of DE genes in each dataset (10%, 30%, and 60%) versus three different numbers of associated studies (two, five, or ten datasets out of 20). The area under the receiver operating curves (AUCs) are compared for the nine scenarios in Fig. 3. The corresponding TPRs and true FDRs are available in Figure S1. The best performing methods were wFisher and Fisher, closely followed by ordmeta. These three methods especially surpassed other methods when only two or five associated studies were included. They also showed the highest TPRs and decent true FDRs. The commonly used FEM and REM showed very low TPRs when only two associated data were included, but that of FEM increased rapidly as more associated datasets were included. As the number of associated studies increased to ten, REM and FEM also showed high AUCs closely following the proposed methods. The proportion of DE genes did not show a high impact on most methods except for Stouffer’s method which dropped noticeably as more DE genes were included. Overall, these results show that the two proposed methods as well as Fisher’s method are able to detect incomplete associations effectively using only the *p*-value information.

### Meta-analysis of microarray gene expression data

The performance of the same eight meta-analysis methods were compared using the nine prostate cancer microarray datasets available from metaDE R package. The true and false DE genes were selected using the six “good” datasets and the incomplete association was simulated by permuting the sample labels of seven datasets as described in “Methods”. These real data-based simulation results were very similar to the model-based simulation results shown in the previous section. The best AUCs and TPRs were obtained by wFisher closely followed by Fisher and ordmeta (Fig. 4). Most methods showed good true FDRs except for rankProd (Figure S2). roP was tested for three order parameter values *r* = 2, 4, or 6. roP_2 and roP_4 showed a good performance but roP_6 performed much worse. This shows the order parameter *r* has a critical effect on the performance of roP; however, the appropriate value of *r* is not known in advance. In summary, wFisher, Fisher and ordmeta outperformed other meta-analysis methods in detecting incomplete associations in both model-based and real data-based simulation tests.

**Figure 5:**
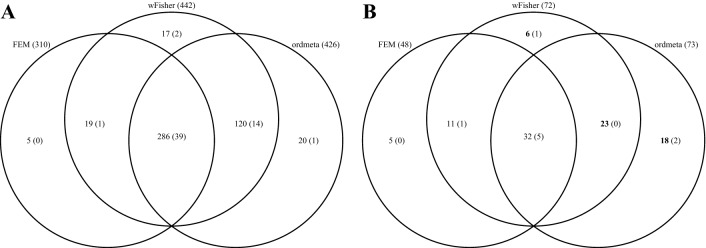
Distribution of significant genes for three meta-analysis methods: fixed effects model, ordmeta, and wFisher. Counts of genes are shown (**A**) including and (**B**) excluding significant genes found from individual studies. All the nine datasets were used for the meta-analysis. *Q*-value of 0.01 was used to select significant genes. Counts of gold standard genes (C0376358 maligant neoplasm of prostate geneset) were shown in the bracket.

We then analyzed the original nine microarray datasets to compare biological relevance of the meta-analysis results. The counts of DE genes detected by each of three meta-analysis methods (wFisher, ordmeta, and FEM) are shown in Fig. 5A. The 676 genes that belonged to all the nine datasets included 67 gold standard genes in DisGeNET database (malignant neoplasm of prostate geneset). We then evaluated the relative ratio of gold standard genes within DE genes in each meta-analysis method by enrichment *p*-values and corresponding ORs (Table 2). Although FEM showed significant enrichment of gold standard genes within its DE genes (*p*-value 0.0395, OR = 1.55), wFisher and ordmeta exhibited even higher significance and ORs (wFisher *p*-value = 0.0048, OR = 2.22; ordmeta *p*-value = 0.0076, OR = 2.04), demonstrating increased biological relevance of the DE analysis results. This result was rather expected because the nine datasets included the three “bad” datasets constituting the condition of incomplete association. Thus, we also compared the biological relevance using only the six “good” datasets. The difference between the methods were much reduced as expected, but wFisher and ordmeta still showed better results (wFisher *p*-value = 0.00026, OR = 1.51; ordmeta *p*-value = 0.00062, OR = 1.46) compared to FEM (*p*-value 0.0013, OR = 1.41). This implies even when only qualified data are used for meta-analysis, each gene can have different association patterns for the individual studies because of the heterogeneity of the populations and other study-specific factors.

Then, we removed all the meta-analysis DE genes that the analysis of individual study can also detect. This uncovers the true ability of each meta-analysis method to discover new genes (Fig. 5B). As compared to FEM result, wFisher and ordmeta additionally found 29 and 41 significant genes, respectively, whereas FEM found 5 and 16 significant genes that wFisher and ordmeta missed, respectively. The evidence from the literature for 25 out of the 47 genes additionally found by wFisher or ordmeta relevant to prostate cancer are summarized in Table S1. For example, three genes (SOD2, U2AF1, and M6PR) out of the 25 genes belonged to gold standard genes (C0376358). SOD2 is a strong antioxidant enzyme, reported to exert roles in damage caused by reactive oxygen species which induce DNA damage and promote oncogenic transformation by increasing mutation rate. Plymate et al.^31^ demonstrated that SOD2 takes a role in suppressing prostate tumor by cell cycle interruption and apoptosis. U2AF1 is a splicing factor subunit that showed an association with various cancer types. Cao et al.^32^ suggested U2AF1 is associated with prostate cancer by regulating androgen receptor variant 7 (ARV7) splicing which is well known to promote proliferation and metastasis of prostate cancer. M6PR was related to apoptosis and proliferation, and differentially expressed in androgen-independent prostate cancer compared with androgen dependent prostate cancer^33^. IGF1R and ISNR were not included in the C0376358 gene set, but were reported of the oncogenic functions including tumor growth, cell migration and angiogenesis in prostate cancer^34^.

### Meta-analysis of BMI association data

The summary statistics of exome-based association study for BMI comprising 246,328 low-frequency and rare variants (GIANT consortium^35^) were analyzed for four ethnic groups (African, Hispanic, Eastern, and South Asian). The European data were excluded in this test because of its dominant sample size. For the four summary datasets, eight different meta-analysis methods such as ordmeta, wFisher, Fisher, Stouffer, RE-HE, MR-MEGA, fixed and random effects models were applied. The loci with *p*-value < 2.03E−7 (= 0.05/246,328) were regarded significant. In total, 27 loci were detected significant by at least one method (Table 3, Supplementary Table S2). Fixed effects model and recently developed MR-MEGA were the most powerful and detected eight and nine loci with the most significant *p*-values between the eight methods. However, ordmeta also detected four loci with the most significant *p*-values. For example, rs12243326 (the 6th, Table S2) had *p*-values of individual cohorts, 0.71, 0.39, 2.80E−06, and 6.50E−06. The former two were far from significant, but ordmeta detected the second smallest 6.50E−06 as the optimal and yielded the most significant combined *p*-value of 2.03E−09, whereas the fixed and random effects models failed to detect this incomplete association. In particular, rs987237 (the last) was detected significant only by ordmeta, because the third individual *p*-value 1.85E−03 was significant as assessed by joint distribution of order statistic. This locus was detectable by neither individual study nor any of the existing meta-analysis methods because of the large last *p*-value of 0.95. The ordmeta *p*-values for the first two loci were 0 (< 1E−323), because the third individual *p*-values 1.70E−06 and 1.40E−06 had extremely small marginal *p*-values. wFisher also detected three loci with the most significant *p*-values, whereas Fisher’s method detected none. These results indicate that ordmeta and wFisher are complementary to the conventionally used meta-analysis methods (e.g., Fisher, fixed, and random-effects models), and demonstrate their potential to provide additional findings.

**Table 3:** Seven loci detected with the most significant *p*-values by either ordmeta or wFisher. Significant *p*-values (< 2.03E−07) were bold faced, and the most significant value between the eight methods compared were underlined. The *p*-values for individual cohorts were synchronized to the effect direction. See Table S2 for the original two-tailed *p*-values for individual cohorts and the full comparison results for the 27 significant loci.

Loci	*p-*values for individual cohorts (synchronized)				Combined *p*-values				
	African	East Asian	Hispanic	South Asian	Ordmeta	wFisher	Fixed effects	Random effects	MR-MEGA
rs1558902	8.50E−07	2.00E−03	1.20E−08	2.95E−13	**0**	**6.16E−25**	**3.92E−26**	**5.08E−26**	**4.78E−25**
rs1421085	7.00E−07	2.00E−03	9.50E−09	7.50E−13	**0**	**1.01E−24**	**6.76E−26**	**7.19E−24**	**8.32E−25**
rs7903146	2.60E−04	0.59	1.95E−06	2.80E−08	**1.82E−10**	**7.92E−14**	**9.21E−14**	6.48E−06	**2.83E−13**
rs489693	3.05E−02	9.00E−04	4.10E−02	2.30E−09	**7.34E−08**	**3.41E−11**	**1.67E−10**	0.000575	**3.52E−11**
rs2206277	1.15E−06	0.83	3.00E−04	3.35E−03	1.19E−06	**2.95E−09**	**4.79E−08**	0.022122	**1.10E−08**
rs12243326	0.71	0.39	2.80E−06	6.50E−06	**2.03E−09**	**4.40E−08**	4.01E−05	0.094958	**1.16E−08**
rs987237	3.35E−04	0.95	3.55E−04	1.85E−03	**2.02E−07**	3.64E−07	3.49E−06	0.073536	2.37E−06

## Discussion

In this article, we focused on the hypothesis of meta-analysis that one or more studies involved are associated, whereas conventional meta-analyses have assumed all or most of the studies are associated when testing their performance. The former tests the ‘existence’ of association, while the latter tests the ‘dominance’ of association among the studies. This difference is reminiscent of the arguments between self-contained and competitive approaches in gene-set analysis which is applied to the dimension of genes^36,37^.

We showed in this study that many existing meta-analysis methods, such as random-effects, fixed effects, *Z*-method, and Lancaster’s method are considerably affected by presence of unassociated statistic values. However, the classical Fisher’s method exhibited relatively high power when unassociated *p-*values were present. Because experimental data can yield unassociated statistic for many reasons, the robustness of Fisher’s method warrants further consideration in meta-analysis. Interestingly, the Lancaster’s method, despite being a weighted version of Fisher’s method, drastically lost its power upon addition of unassociated statistics. Lancaster’s method uses large weights for its DFs such as the sample sizes for each experiment. These large DFs make individual distributions close to normal distribution, which causes a power decrease similar to that of *Z-*method. In contrast, the classical Fisher’s method uses DF of only two for each experiment, keeping individual distributions right-skewed. This feature makes Fisher’s method highly sensitive, even when only a small number of *p-*values are associated. Therefore, we proposed to use a generalized Fisher’s method with non-integer weights where DFs are given proportional to sample sizes, while the total sum of DFs is kept as small as that of Fisher’s method. This yielded a generally useful *p*-value combining method, wFisher, which exhibited improved power, both with or without unassociated *p-*values. We also proposed another robust *p-*value combining method based on joint distribution of order statistic. Ordmeta focused on the optimal marginal *p-*value and explicitly evaluated its significance without using heavy empirical computation^38^. Ordmeta only relies on the optimal marginal *p*-value; therefore, it is not affected by other large *p*-values. Thus, ordmeta performed relatively well when some bad (large) *p*-values were present.

The two proposed methods, wFisher or ordmeta surmounted existing meta-analysis methods in detecting incomplete associations through several different simulation tests. In particular, they were able to detect many novel genes that existing methods missed, and also exhibited high biological relevance with or without unassociated data. This implies a substantial number of genes are associated in only a subgroup of studies in meta-analysis. We note that *p*-value combining methods are only able to address the significance of association (or differential expression), but not the effect size. However, we demonstrated the usefulness of some *p*-value combining methods including ours. This does not mean that our methods are more powerful than the conventional methods. Indeed, conventional meta-analysis methods were more powerful than ours for the genes that are associated in most studies under consideration. Therefore, our methods and conventional methods are complimentary to each other, and can be used together to maximize our findings in meta-analysis.

Besides, it is also an important challenge to integrate the evidences from different types of medical and genomic data where many of them could be unrelated to the disease of interest. *P*-value combining methods are generally useful when combining heterogeneous types of data irrespective of the data models and statistical methods applied in each study. We expect the proposed methods will be able to provide many new findings in meta-analyses of gene expression, GWAS, and other genomic and medical data.

## Supplementary Information

Supplementary Figures.

Supplementary Table S2.

Supplementary Table S1.
