# Supplementary material for: Powerful p-value combination methods to detect incomplete association
Source: Sci Rep. 2021 Mar 26;11:6980. doi: 10.1038/s41598-021-86465-y (PMC7997958; doi:10.1038/s41598-021-86465-y)
Supplement: Supplementary file 1 — Supplementary Figures. [file 41598_2021_86465_MOESM1_ESM.docx]

Powerful *p*-value combination methods to detect incomplete association

Sora Yoon^1^$\dagger$ , Bukyung Baik^1^$\dagger$, Taesung Park^2,3^, Dougu Nam^1,4*^

^1^Department of Biological Sciences, Ulsan National Institute of Science and Technology, Ulsan 44919, Republic of Korea

^2^Department of Statistics, Seoul National University, Seoul 08826, Republic of Korea

^3^Interdisciplinary program in Bioinformatics, Seoul National University, Seoul 08826, Republic of Korea

^4^Department of Mathematical Sciences, Ulsan National Institute of Science and Technology, Ulsan 44919, Republic of Korea

$\dagger$ These authors contributed equally to this paper

*To whom correspondence should be addressed. Tel: +82-52-217-2525; Fax: +82-52-217-2639; Email: dougnam@unist.ac.kr

**Figure S1. RNA-seq simulation results.** (A) TPR and (B) trueFDR of eight meta-analysis methods were compared for different numbers of associated studies and significant genes. Among 20 studies used for the meta-analysis, 2, 5 and 10 studies were introduced as associated studies. The results with 10, 30 and 60% of significant genes are shown in the first, second, and third rows, respectively.

**Figure S2. Microarray real data results.** (A) TPR and (B) trueFDR of ten meta-analysis methods were compared. The sample labels of seven out of the nine studies were permuted to make unassociated studies. roP was tested for three *r* parameters, 2, 4, and 6 denoted as (roP_2, roP_4, and roP_6, respectively).
